# Implementation of Group and Individual Supervision Techniques, and Its Effect on the Work Motivation and Performance of Teachers at School Organization

**DOI:** 10.3389/fpsyg.2022.943838

**Published:** 2022-07-22

**Authors:** Bambang Budi Wiyono, Sulis Peni Widayati, Ali Imron, Abdul Latif Bustami, Umi Dayati

**Affiliations:** ^1^Department of Educational Administration, Faculty of Education, Universitas Negeri Malang, Malang, Indonesia; ^2^Nonformal Education Study Program, Faculty of Education, Universitas Negeri Malang, Malang, Indonesia; ^3^Sociology Education Study Program, Faculty of Social Science, Universitas Negeri Malang, Malang, Indonesia

**Keywords:** group supervisions, individual supervisions, work motivation, performance, elementary school teachers

## Abstract

Teachers have a very important role in determining the quality of the teaching-learning process and the students’ learning outcomes. Learning outcomes will optimally be achieved if it is supported by qualified teachers. One way to enhance the teachers’ performance is through instructional supervision which can be divided into two techniques, namely group and individual supervision techniques. Therefore, this study aims to find out the influence of instructional supervision techniques on the work motivation and performance of elementary school teachers. This study was conducted in East Java, Indonesia, and an explanatory research design was used. The sample was taken from 80 elementary school teachers in Malang and Blitar using a multi-stage random sampling technique. Data were collected through the use of questionnaires and documentation, and then they were analyzed by using the structural equation modeling technique. The result of this study showed that group supervision has a significant effect on teachers’ performance, whereas individual supervision influenced teachers’ work motivation and it affected their performance.

## Introduction

Teachers are the key to the educational system. Good quality of the learning process will be achieved if teachers can perform their roles professionally. Moreover, students learning outcomes will also be achieved optimally if teachers can apply a high-quality learning process. Characteristics, experience, and pedagogical content of the knowledge of teachers influence students’ proficiency development and achievement (Goldhaber and Starz, 2017; Koopman et al., 2017). In addition, teachers’ acceptance, teachers’ support, and teacher-student relationship influence the students’ achievement (Košir and Tement, 2014). Teachers’ motivation also affects student achievement. The teachers’ motivation mediation between teacher enthusiasm and student achievement (Valentín et al., 2022). Teachers’ autonomous motivation play a sequential mediator in the link between informal learning and innovative teaching performance during the COVID-19 pandemic (Yu et al., 2021). Therefore, to increase the education quality of schools, teachers’ professionalism should always be improved. One of the ways to increase the teachers’ professionalism is through supervision.

Supervision is the process of assisting teachers, for instance, through guidance, stimulation, consultations, or other teachers’ development programs to improve their skills in accomplishing their tasks. Consequently, there are various teaching assistances for example guidance done by supervisors, principals, or other instructors to enhance the teaching and learning process. In addition, there are two main goals in implementing the supervision, to improve teachers’ skills and their work motivation to enable them to carry out their tasks. Proctor and Inskipp identified three broad purposes of supervision, which have been widely accepted, namely monitoring the quality of professional services, enhancing professional competencies, and supporting the supervisee to express, conduct, and reflect on their work (Beinart and Clohessy, 2017). The study result of the work of McMeeking et al. (2012) showed that students’ odds of achieving a score of proficient or better increased with teacher participation in the professional development program. The professional development program is one of the forms of supervision.

## Review of Literature

### Instructional Supervision

In carrying out instructional supervision, supervisors need to apply suitable principles, techniques, and approaches. Several principles should be followed when carrying out supervision. Those principles are divided into two, namely the positive and negative principles. The positive principles are those that have to be completed, for example constructive, creative, cooperative, democratic, and refer to the objectives. They should also be based on professional relationships, done systematically and objectively, and also develop teachers’ potency. Whereas, negative principles are the ones that should be avoided such as those who are not on the track, not only looking for errors and not based on the authority.

Besides the principles, there is another aspect that needs to be considered, namely the supervision approaches. Glickman et al. (2007) presented three supervision approaches; they are directive supervision, non-directive supervision, and collaborative supervision. On the other hand, the supervision approach is completed through supervision techniques which have different methods of doing supervision. Supervision techniques are divided into two categories based on the number of the teachers, namely group and individual techniques. Group techniques are conducted if there are a lot of teachers that are supervised. These include teachers’ meetings, teaching demonstrations, workshops, seminars, training, and discussions, while individual techniques are conducted when the supervision is carried out for a teacher individually. Some examples of them are classroom visitation, self-evaluation, classroom observation, and individual conferences. Furthermore, the supervision technique also can be classified into two; namely direct and indirect techniques. Direct techniques are carried out when there are direct communications between supervisors and teachers such as workshops, classroom visitation, and teachers’ meetings. While indirect techniques are held through media for instance supervision bulletins.

Based on the theory, it appears that the main purpose of supervision is to improve the professionalism of teachers in carrying out their duties. However, the objective has not been achieved, both in the process and in the result. The study results of Yavuz (2010) showed that supervisors in the process of supervising teachers have not conducted the correct behaviors in line with the principles of supervision. That means that many supervision activities have not shown a good process and result.

The objective of the supervision is to improve the professionalism of teachers in carrying out their professional tasks, whereas it is still not achieved optimally. Based on the review team report of National Policies for Education, most teachers also do not experience regular ongoing professional development training and there is very little on-the-job coaching provided by external facilitators, principals, supervisors, or experienced teachers (OECD and Asian Development Bank, 2015). In addition, the study results of Wiyono (2016) showed that the supervision techniques in Indonesia have not been implemented at schools intensively. The process of supervision implementation did not refer to the supervision principles comprehensively as well. Several supervision techniques are still very poorly implemented, including collegial supervision, lesson study, professional libraries, and school visitation. Therefore, it is necessary to research the effectiveness of these supervision techniques, both group supervision techniques and individual supervision techniques, to improve teacher professionalism.

### The Relationship Between Instructional Supervision, Work Motivation, and Performance of Teachers

The professionalism of teachers is based on two aspects, namely work motivation and performance. Professional teachers have high work motivation and performance when performing their tasks. Glickman et al. (2007) called these aspects the level of commitment and level of abstraction. The level of commitment refers to motivation, while the level of abstraction refers to competence which is manifested in the teacher’s performance. Therefore, the achievement of supervision objectives can be assessed from the increase in the teachers’ work motivation and performance.

Motivation is derived from the Latin word, “movere” meaning “move,” or the English word “motive” which implies impulse. The motive could be described as a power within a person that encourages one to do something. Motivation is a motive that is activated. Thus, work motivation is an internal drive that gives strength, direction, and resilience to individuals to conduct tasks in certain ways. An individual who has high work motivation will carry out a task in earnest. In contrast, someone who has low work motivation will not carry out the task seriously.

Performance is an action or a result of work achieved by personnel in carrying out their tasks. In the program of education, the results can be classified into two, namely output and outcome of education. Students’ achievement, results of the teaching and learning process, and parents’ satisfaction are the educational outputs. The success of school graduates studying at a higher educational level or working in the community indicates the educational outcomes.

Several factors influence the motivation and performance of teachers. School conditions that meet the psychological needs have a positive effect on teacher motivation. Teachers with more autonomy support will have higher satisfaction and stronger teaching motivation, which further enhances their work engagement (Zhang et al., 2021). Teacher work engagement affects student academic achievement, especially for the teachers with high self-efficacy (Wang, 2022). The principals’ leadership behavior or styles also affects the motivation and performance of teachers (Parveen et al., 2022). Likewise, behavior in the implementation of supervision also affects teacher motivation and performance (Schyns et al., 2018).

Based on the theory, there is a very strong relationship between instructional supervision and motivation and the work performance of the teacher. Supervision objectives can be achieved if it improves the work motivation and performance of teachers. Several previous types of research show different results. Packard and Dereshiwsky identified one of the main factors that determine teacher work motivation as leadership and teacher development (Han and Yin, 2016). In line with these findings, the research results of Wiyono and Triwiyanto (2018) showed that there was a significant relationship between teachers’ participation in the teacher working group meetings and their professionalism. Teacher working group meeting is a group supervision technique. Moreover, another study by Wiyono et al. (2017b) which was applied to school principals showed that self-evaluation influenced the principal’s transformational leadership, which has an impact on increasing work motivation and teacher work effectiveness. Self-evaluation is a type of supervision that focuses on the individual’s development.

In addition, several previous studies showed different results. Wiyono et al. (2017a) presented that several supervision techniques affected teachers’ performance. Those techniques did not differentiate between individual and group techniques. Moreover, another study by Wiyono et al. (2017b) which applied to school principals showed that self-evaluation influenced the principal’s transformational leadership. Self-evaluation is a type of supervision that focuses on the individual’s development. However, it is still unclear how the comparative effect is with group supervision.

In the other study, Abdullah et al. (2009) proved that there is a correlation between teacher training and their productivity. The results of research by McGregor and Gunter (2001) showed that teacher professional development programs could improve teacher competence. The result of the study done by Reed et al. (2002) presented that a self-reflection program was carried out as a tool to enhance teachers’ professionalism as well. Teacher training is one example of group supervision techniques, and the self-reflection program is one of the individual supervision techniques.

In contrast, other studies have shown that there is no influence of the supervision techniques on the teachers’ performance. Shah et al. (2011) showed that in-service education did not affect the teachers’ performance. The results of Sahbaz (2011) study also showed that the in-service training program, which was a supervision technique, did not show a significant effect on the performance of the counselor. The study results of Wiyono et al. (2017a) also showed that out of the 25 supervision techniques, it was only eight techniques that showed a significant influence on the teacher’s work performance. Those techniques were not differentiated from the individual and group techniques. Thus, there are only a few effective supervision techniques to improve teacher performance. The research results of Lombardi (2001) also showed that the supervision techniques applied today do not affect improving teachers’ competency. For this reason, it is necessary to find innovative supervision techniques that are more effective in improving teachers’ competencies, motivation, and professionalism.

Based on the results of several studies, it can be concluded that the implementation of some supervision techniques shows different research results. The characteristics of behaviors applied in the implementation of individual supervision and group supervision are different. It will have a different impact on the reaction, satisfaction, motivation, or performance of teachers (Qian et al., 2017; Shen et al., 2020). Hence, those various supervision techniques will provide more opportunities for teachers to develop themselves. However, supervisors should find the most effective technique to help teachers improve their motivation and competencies. Therefore, it is necessary to find effective supervision techniques, group techniques, or individual techniques, that can improve the teachers’ work motivation and performance. How the structural relationship model of supervision techniques, teachers’ work motivation, and performance needs to be studied more deeply. For this reason, this study is being conducted.

The objectives of this research are (1) to find out the effect of group and individual supervision techniques on the elementary school teachers’ work motivation and performance, (2) to discover the most effective supervision techniques to increase elementary school teachers’ work motivation and performance based on the classifications of the supervisions, and (3) to find out the structural effect of the frequency in participating group and individual supervision techniques on the elementary school teachers’ work motivation and performance. The hypothesis formulation that will be tested is (1) there is a significant effect of group and individual supervision techniques on work motivation and performance of teachers, either directly or indirectly, and (2) the teachers’ motivation influence the teachers’ performance.

## Materials and Methods

### Research Procedure

This study was held in Malang and Blitar, East Java, Indonesia. Both cities were taken as samples using a multistage random sampling design. First, two cities from many cities in East Java as samples randomly. Then, 40 elementary schools in each city were randomly taken as samples, and 80 teachers were randomly chosen as the analysis units. The explanatory research design was used combined with the equation structural modeling analysis technique. This study used explanatory design because researchers aim to test hypotheses and theories that explain how and why a phenomenon operates as it does. In other words, the researchers established evidence for the cause and effect of the relationship between these phenomena (Johnson and Christensen, 2004). The researchers explained the structural relationship between supervision, teacher’s work motivation, and performance, either directly or indirectly, both as latent variables and manifest variables. Thus, a comprehensive conclusion was to be obtained.

### Research Instruments

Based on the objectives of this study, there were four main variables tested; namely (1) the group supervision technique, (2) the individual supervision technique, (3) work motivation, and (4) teachers’ performance. Questionnaires were used to collect the main data in this research, and it was completed using documentation. Meanwhile, the research instrument was developed based on the research variables. The instrument model used a scoring scale, a Likert scale (summated rating), and opened questionnaires.

In addition, the scoring scale was used to collect the group and individual supervision data. The group supervision was shown in two dimensions (observed variables), namely (a) the group supervision done by supervisors for example trainings, seminars, workshops, teaching demonstrations, and so on and (b) the collegial group supervision techniques for instance lesson study, action research, discussion with peers, etc. Moreover, the individual supervision was also presented in two dimensions, namely (a) the individual supervision completed by supervisors such as clinical supervisions, classroom visitation, individual conferences, etc., and (b) the self-directed supervision for example supervision bulletins, library, and self-development.

Likert scale (summated rating) was used to measure teachers’ work motivation which was shown in two dimensions (observed variables); namely (a) the motivation in accomplishing teaching and learning tasks for example writing lesson plans, conducting teaching process, completing the learning evaluation, providing remedial teaching and enrichment lessons, etc.; and (b) the completion of professional development tasks are writing a thesis, conducting research, writing papers for publication, producing course books, developing teaching aids, completing the class administration, and so on.

Correspondingly, the performance of teachers was measured using opened and closed instruments which were then shown in two dimensions (observed variables), namely (a) the achievement of the work based on the standard set and (b) the achievement of the work according to the target set by teachers, and the expectation of parents and the society. Then, a trial was carried out to test the instrument before it was used in this research. Consequently, the analysis result of the trial showed that the three instruments used were valid. The instrument of the supervision technique was valid with the loading factor of >0.3 for each item, while the reliability of the Cronbach coefficient was 0.898 (> 0.7). The instrument of the work motivation was also valid with the loading factor of >0.3 for each item, while the reliability of the Cronbach coefficient was 0.895 (> 0.7). In addition, the instrument of the teacher’s performance was valid with the loading factor of >0.3 for each item, while the reliability of the Cronbach coefficient was 0.817 (> 0.7). Hence, it can be concluded that the research instruments were valid and reliable.

### Data Analysis

According to the objectives of this study and the available type of data, data analysis techniques used were descriptive statistics and inferential statistics. The descriptive statistics used in this research were mean, distribution frequency, percentage, and graphical method, whereas the inferential statistics used Pearson product–moment correlation and structural equation modeling. The structural equation modeling was analyzed using Lisrel (Linear Structural Relation) program. It was used to find the dimensions of variables, and relationships among the variables, and also to examine the structural effects of the exogenous variables on the endogenous variables or the endogenous variables on the endogenous variables, directly or indirectly.

## Results

### Implementation of Group and Individual Supervision Techniques

The main objective of this study is to find out the effect of teachers’ participation in the following group and individual supervision techniques on teachers’ work motivation and performance. Besides that, through this study, the comparison of the effect of group and individual supervision on the teachers’ work motivation and performance would be obtained. Before the hypothesis of the test result was presented, the frequency of teachers participating in group and individual supervisions would be described first.

The group supervisions are classified into two categories, namely (1) group supervisions done by supervisors including teacher meetings with supervisors, trainings, seminars, teaching demonstrations, “in-on-in” activity programs, seminars and workshops, and courses and (2) collegial supervisions which include teacher working group meetings, discussion with peers, classroom intervisitation, lesson study, action research, teaching simulation, school intervisitation, comparative studies, and peer supervision. Furthermore, individual supervisions are classified into two categories, namely (1) individual supervisions done by supervisors which include clinical supervisions, classroom visitation, new teacher orientation programs, and individual conferences and (2) self-directed supervisions which cover supervision bulletins, supervision library, and self-development.

Based on the data analysis, it shows that most teachers often participate in group supervision done by supervisors with a mean score of 2.13, then collegial group supervision with a mean score of 2.05, individual supervision done by supervisors with a mean score of 1.58, and the last one is self-directed supervisions with the mean score of 0.56.

Based on the data analysis in detail, it can be known that, respectively, the group supervisions done by supervisors include teacher meeting with supervisors, with a mean frequency of 4.04 ( $\bar{x}$ = 4.04), training ( $\bar{x}$ = 2.99), seminar ( $\bar{x}$ = 2.76), teaching demonstration ( $\bar{x}$ = 2.64), in on in activities ( $\bar{x}$ = 1.94), upgrading ( $\bar{x}$ = 1.85), workshop ( $\bar{x}$ = 1.12), seminar-workshop ( $\bar{x}$ = 1.05), and course ( $\bar{x}$ = 0.55). The collegial group supervisions include teacher working group meetings ( $\bar{x}$ = 4.44), discussion with peers ( $\bar{x}$ = 3.36), classroom intervisitation ( $\bar{x}$ = 1.88), lesson study ( $\bar{x}$ = 1.87), action research ( $\bar{x}$ = 1.77), teaching simulation ( $\bar{x}$ = 1.73), school intervisitation ( $\bar{x}$ = 1.46), comparative study ( $\bar{x}$ = 1.27), and peer supervision ( $\bar{x}$ = 1.08). Then, the individual supervisions done by supervisors include classroom visitation ( $\bar{x}$ = 2.31), clinical supervision ( $\bar{x}$ = 1.88), individual conference ( $\bar{x}$ = 1.71), and new teacher orientation programs ( $\bar{x}$ = 0.49). Lastly, the self-directed supervisions include self-development ( $\bar{x}$ = 1.68), supervision bulletin ( $\bar{x}$ = 0.41), and supervision library ( $\bar{x}$ = 0.40).

According to the result of the analysis, it can be concluded that most elementary school teachers in Indonesia participate in group supervision more than individual supervision. In the group supervision done by supervisors, the techniques mostly followed by teachers are teacher meetings with supervisors, trainings, seminars, and teaching demonstrations, while in the collegial group supervision, the techniques mostly followed by teachers are teacher working group meetings and peer discussions. In contrast, a lot of teachers rarely participate in individual supervision, especially in self-directed supervision. In other words, teachers still do not get used to develop themselves independently.

### The Effect of Instructional Supervision on the Teacher’s Work Motivation and Performance

According to the result of the analysis, it could be seen that the result of the structural equation modeling analysis of the Chi-square obtained was 16.752 with *p* = 0.402. The *p* > 0.05, consequently, it can be concluded that the hypothesis model which was formulated in this study fit into the actual data. The results are proven by some other criteria of goodness of fit which are presented in Table 1.

**Table 1:** The analysis results of the goodness of fit of structural relationship model among variables.

Indicators of goodness of fit	Output	Criterion	Interpretation
Chi-square	16.752, *p* = 0.378	*p* > 0.05	Fit
Good of fit (GFI)	0.950	> 0.9	Fit
Root-mean-square residual (RMSEA)	0.024	< 0.05	Fit
Normed fit index (NFI)	0.952	> 0.9	Fit
Non-normed fit index (NNFI)	0.994	> 0.9	Fit

**Figure 1:**
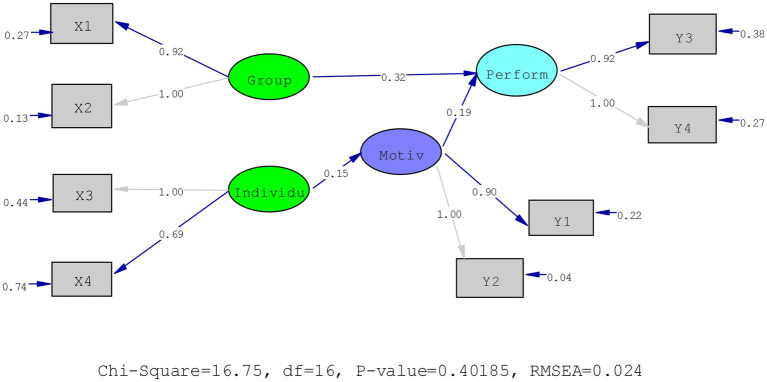
Relationship between exogenous variables and endogenous variables. Group, group supervision technique; X1, group supervision by supervisor; X2, collegial group supervision; Individu, individual supervision technique; X3, individual supervision by supervisor; X4, self-directed supervision; Motiv, teachers’ work motivation; Y1, motivation in doing teaching and learning tasks; Y2, motivation in doing professional development tasks; Perform, teachers’ performance; Y3, the achievement of the work result based on the standard set; Y4, the achievement of the work result based on the expectation.

Based on the scores of the criteria of goodness of fit shown in Table 1, it can be concluded that the hypothetical model of correlation between exogenous and endogenous variables proposed in this study fits into the real data obtained. Hence, the null hypothesis is rejected and the alternative hypothesis is accepted. In general, the structural relationship model among those variables is presented in Figure 1.

In Figure 1, it was presented that the relationship model between exogenous and endogenous variables and between endogenous and endogenous variables fit with the real data is observed from the Chi-square score, the goodness of fit (GFI), Root-mean-square residual (RMSEA), Normed fit index (NFI), or Non-Normed Fit Index (NNFI). The frequency of teachers participating in group supervision directly affects their performance as well as their work motivation. In contrast, it does not affect the teachers’ performance. Moreover, teachers’ work motivation directly affects their performance.

Based on the results of the data analysis, it can be concluded that the frequency of teachers participating in group supervision directly influences teachers’ performance with a coefficient of 0.317 and the *t*-value is greater than the critical value. The more active teachers participate in group supervision, the better results will be achieved by the teachers. While individual supervision influences work motivation with a coefficient of 0.154, there is a direct effect between teachers’ work motivation and the results achieved with the coefficient of 0.191 and the t-value is higher than the critical value.

Based on the data analysis in detail, it can be known that the obtained score for group supervision by supervisors is *λ* = 0.853; and the obtained score for collegial group supervision is *λ* = 0.931. Both Lambda scores are quite high; therefore, it shows that both observed variables are the main indicators that construct the group supervision. Based on the analysis results, it is proven that the score obtained in individual supervision done by supervisors is *λ* = 0.750; and the self-directed supervision is *λ* = 0.514. Both Lambda scores in these categories are also high; consequently, they showed that the observed variables above are the key indicators that construct the individual supervisions.

Based on the data analysis, it is also proven that the obtained score in the motivation to complete the teaching tasks is *λ* = 0.885; and the score in the motivation to achieve the professional development is *λ* = 0.979. Both Lambda scores are high as a result both observed variables are the key indicators that construct teachers’ work motivation. Based on the analysis results, it was also shown that in the performance variables, the score obtained in the achievement of work based on the standard set is *λ* = 0.788; and the score obtained in the achievement of work based on the expectation is *λ* = 0.845. Both Lambda scores in these categories are also high; therefore, it can be concluded that the observed variables are the main indicators that construct teachers’ performance.

## Discussion

### Implementation of Instructional Supervision Techniques

Based on the data analysis, it can be concluded that most teachers participate in group supervision more than individual supervision techniques. The result of this study is in line with the study conducted by Wiyono (2016) which showed that some supervision techniques followed mostly by teachers were teacher meetings, teacher working group meetings, peer discussions, and upgrading. The four supervision techniques are group supervision techniques. Most teachers participate in the group supervisions organized by supervisors, and they have not conducted self-development independently.

### The Effect of Group Supervision Techniques on Teacher’s Work Motivation and Performance

The second result of this study shows that there is a direct effect between teachers’ participation in group supervision and their performance. This finding is in line with previous studies. Wiyono and Burhanuddin (2015) have shown that teachers’ group meetings influence the quality of teachers’ performance in the teaching and learning process. Moreover, the result of this study is also in line with the research done by Malm (2009) which stated that the training programs improve teachers’ cognitive, emotional, and social competencies. Rahman et al. (2011) have also shown that there is a correlation between teacher training and teaching effectiveness. Furthermore, the study done by Gersten et al. (2010) has also proven that teachers’ study group influences their knowledge, especially their teaching skills and teaching practices. Shah et al. (2011) have presented that in-service education and training programs positively affect teachers’ performance in managing the class, using various teaching methods, producing teaching aids, and evaluating techniques. The study results of Somers and Sikorova (2002) also show that teachers’ in-service education program can improve the teaching practice of teachers. In addition, the study by Yigit (2008) has shown that in-service training programs improve teachers’ attitudes toward the use of technology in the teaching and learning process. In line with previous studies, Bisset and Nichol (1998) also found that special courses influence the teachers’ way of thinking, teaching practice, and professionalism. Some examples of group supervision techniques are trainings, teachers’ group meetings, courses, and in-service training programs.

The other research conducted by Alsaleh et al. (2017) showed that the peer coaching strategy fosters pre-service teachers’ professional development growth. The peer coaching strategy is one of the group supervision techniques. The study results of Maisyaroh et al. (2017) also indicated that there is a significant positive correlation between the frequency and principles of supervision and teachers’ teaching skills. Therefore, this research has supported the results of the previous studies that group supervisions significantly influence teachers’ performance.

Furthermore, it can be understood that group supervision techniques are more effective to enhance teachers’ performance than individual supervision techniques since, in group supervision, teachers can interact and provide positive feedback to their peers. Through the interaction, teachers are motivated to participate during the supervision process. This is in line with the study of Wiyono et al. (2017a) which shows that among eight supervision techniques that significantly and positively correlate with teachers’ performance, six of them are group supervision techniques and only two are individual supervision techniques. Another research done by Wiyono et al. (2015) has also proven that collaborative supervision techniques emphasize the activeness of teachers in participating in the supervision and are also based on humanistic principles. On the other hand, the literature review conducted by Stark et al. (2017) also indicated that collaborative supervision was very effective to promote teachers’ growth. In addition, the study of Rasyad et al. (2019) also indicated that the gradual training could improve the teachers’ competence in early childhood education. Gradual training is one of the group supervision techniques.

Group supervision directly influences the performance of teachers; however, they do not have any effect on teachers’ work motivation. Individual supervision techniques have more influence on teachers’ work motivation. Furthermore, group supervision emphasizes more on the improvement and increase of teachers’ performance. Group supervision aims to improve and enhance the quality of the teaching and learning process. The result of the study by Askew et al. (1997) presented that teachers’ participation in professional development programs affects their beliefs and the achievement of students learning outcomes. Gersten et al. (2010) also discovered that there is a significant positive correlation between knowledge and teaching practice and students’ achievement. Therefore, the study results reinforce the previous findings that effective supervision needs to emphasize collaboration and interaction of teachers based on humanistic principles (Lombardi, 2001; Wiyono et al., 2015).

### The Effect of Individual Supervision Techniques on Teachers’ Work Motivation and Performance

The third result of this study shows that the individual supervision technique is more effective in enhancing teachers’ work motivation than the group supervision technique. However, group supervision techniques are proven to be more effective to improve teachers’ performance. The finding is in line with the theory that individual motivation occurred through needs (Kowalsky, 2003). Individual supervision was primarily intended to improve or solve an individual’s problems (Ministry of Education and Culture, 2016). By doing individual supervision, the principal will understand the needs of each teacher, so that he can increase the teacher’s work motivation (Zhang et al., 2021). Therefore, the results of this study are in line with the results of the previous studies and the theory of motivation. In the study of Zhang et al. (2014), it was shown that self-evaluation influences job satisfaction. Self-evaluation itself is one of the independent development programs that is completed by an individual. Furthermore, it is also similar to Wiyono et al. (2017b) which showed that self-evaluation influences the principal’s transformational leadership skills. Similarly, Orpen (1997) proved that formal mentoring programs provide positive effects on teachers’ motivation.

Furthermore, the direct effect of individual group supervision on the teachers’ work motivation is low, that is because the participation of teachers’ in the individual supervision technique is low, so it cannot improve their work motivation. Besides that, the supervisors have not applied the supervision principles comprehensively during the supervision process. The research finding is in line with Rahabav (2016) study which indicated that supervisors did not have sufficient competencies for implementing academic supervision. Based on two data sources, namely principals and teachers, academic supervision has not been done professionally. On the other hand, the study result conducted by Felix (2017) has shown that demography, status, personality, perception, school location, and some other factors influence instructional supervision.

In addition, there are many other factors affecting teachers’ work motivation, namely responsibility, teaching experience, etc. (Bishay, 1996). The finding is also in line with Herzberg’s theory which suggested that besides supervision, some factors contribute to teachers’ work motivation, namely recognition, responsibility, advancement, work itself, growth, work conditions, salary, personal life, status, security, relationship with supervisor, peers, and subordinates. The factors are classified into two factors, namely motivator factors and hygiene factors (Lunenburg and Ornstein, 2000). However, based on the results of the structural equation model analysis, there are several effects of the individual supervision technique on teachers’ work motivation. It is necessary to understand the needs of teachers to increase the magnitude of the influence. Therefore, the first step to conducting individual supervision techniques is to analyze the needs and conditions of teachers.

### The Effect of Work Motivation on the Performance of Teacher

Based on the study results, it is well known that motivation is one of the essential aspects that influences teachers’ performance; hence, it is also in line with several other previous studies in the field of management. Wiyono et al. (2017a) have illustrated that work motivation influences the effectiveness of teachers in the group. The meta-analysis result of Cherian and Jacob (2013) has also proven that there is a significant influence between work motivation and teachers’ performance. Moreover, Elqadri et al. (2015) have presented that despite the leadership style and extra wage, there is a significant relationship between motivation and teachers’ performance. Another study by Jerotich (2015) has also shown that there is a positive and significant correlation between teachers’ motivation and students’ performance. The more motivated teachers are, the better the learning outcomes of the students. Consequently, it can be concluded that the result of this research supports the previous studies done by previous researchers that work motivation positively influence teachers’ performance. Work motivation is the dominant factor that determines job performance (Kwapong et al., 2015; Sudarjad et al., 2015; Meindinyo and Ikurite, 2017; Donkoh and Baffoe, 2018; Zhang et al., 2021).

Moreover, in line with the results of these studies, Han and Yin (2016) revealed that some of the results of the research on motivation also showed that a teacher’s work motivation had a significant effect on his or her teaching effectiveness. Teaching effectiveness is the main indicator of a teacher’s performance. Hughes et al. (1999) also showed that work motivation is one of the main factors that determine teachers’ work performance. The higher the teacher’s work motivation is, the higher their performance is. Thus, it can be concluded that the results of this study support the results of the previous studies about work motivation which has a positive effect on employee work performance (Wiyono et al., 2021).

There are two dimensions of teacher work motivation, namely the motivation in doing the teaching and learning tasks and motivation in doing professional development tasks. In addition, there are two dimensions of the teacher’s performance, namely the achievement of work based on the standard set and the achievement of work based on the expectation. The finding is in line with some previous study results (Wiyono et al., 2017a; Wiyono, 2018). The application of supervision that refers to constructive principles and through systematic procedures will be able to increase teacher motivation and performance (Alfian et al., 2019; Samawi et al., 2019). The results of this study can not only be used to improve teacher performance but also can be used by mentor teachers to develop the competence of teacher candidates. Mentor teachers play an integral role in the development of teacher candidates (Floden et al., 2021).

## Conclusion

According to the result of this research, several conclusions can be drawn. Firstly, most Indonesian elementary school teachers participate in group supervision compared to individual supervision. It could happen since there are a big number of elementary school teachers in Indonesia. Therefore, group supervision is the most common technique conducted in Indonesia.

The analysis result of the structural equation modeling indicated that group supervision affects teachers’ performance. On the other hand, it does not show any effect on teachers’ work motivation. This result proves that group supervisions enable teachers to develop their competency more than their motivation. Therefore, a significant influence on performance is observed. The more often a teacher participates in group supervision, the higher their competency is in completing their tasks. Based on the test result of the measurement model, it was found that teachers’ activeness in completing group supervisions done by supervisors and collegial group supervisions are the main observed variables in teachers’ activeness when participating in group supervisions.

Similarly, another analysis result has also indicated that individual supervision does not significantly influence teachers’ performance since there is an effect that is lower than work motivation. Thus, as the frequency of teachers participating in individual supervision is low, it does not indicate if this technique of supervision could enhance teachers’ work motivation and their performance. Nevertheless, based on these findings, it could be concluded that individual supervisions are more effective in enhancing teachers’ work motivation than group supervisions. According to the result of the measurement model, it could be seen that teachers’ activeness in taking part in the individual supervision carried out by supervisors and their activeness in completing the self-development programs are the main observed variables in individual supervision.

Finally, the last analysis result shows that work motivation influences performance. This finding has strongly proven the results of previous studies and theoretical frameworks in which work motivation significantly affects teachers’ performance. The more motivated the teachers are, the better the results of the achievement are achieved by teachers. According to the result of the measurement model test, it was also found that teachers’ work motivation in carrying out the teaching and learning and professional development tasks are the main dimension (observed variables) of the elementary school teachers’ work motivation.

The research findings contribute to developing the supervision theory. Group supervision techniques are effective in improving the teaching competence of teachers. Through the implementation of group supervision techniques, teachers can interact with each other so that they can exchange knowledge and experiences. In addition, teachers can also participate actively in supervision activities to develop themselves optimally. The individual supervision technique is effective in improving teachers’ morale in performing their work. Through the implementation of the individual supervision technique, the conditions, needs, and problems faced by teachers individually can be known, so that supervisors can improve their work motivation well.

In addition, the research findings contribute to human resource management theory. Supervision is one of the factors that influence teachers’ competence and work motivation; however, to achieve the objectives, the supervision process must be implemented systematically and its principles should be applied comprehensively. The first step in supervision implementation is to analyze the needs and the characteristic of teachers appropriately. Based on the teachers’ needs, a good program can be formulated, and it can be implemented properly. Then, it can be evaluated to see the results of the implementation. Through these steps, the teachers’ professionalism in carrying out their tasks will be improved. The results of this study can be used to improve teacher competence and can also be used to develop the competence of teacher candidates. Future research should explore how the impact of each technique on the students’ achievement. It is recommended that future research use an experimental design.

## Data Availability Statement

The raw data supporting the conclusions of this article will be made available by the authors, without undue reservation.

## Author Contributions

All authors listed have made a substantial, direct, and intellectual contribution to the work and approved it for publication.

## Conflict of Interest

The authors declare that the research was conducted in the absence of any commercial or financial relationships that could be construed as a potential conflict of interest.

## Publisher’s Note

All claims expressed in this article are solely those of the authors and do not necessarily represent those of their affiliated organizations, or those of the publisher, the editors and the reviewers. Any product that may be evaluated in this article, or claim that may be made by its manufacturer, is not guaranteed or endorsed by the publisher.
